# Longitudinal Study and Characterization of Gait Impairment in a Mouse Model of SCA1

**DOI:** 10.1007/s12311-025-01910-2

**Published:** 2025-09-18

**Authors:** Siddhartha Maharjan, Eliyahu Kochman, Tatiana Gervase, Nina Page, Mannut Singh, Rajveer Singh, Avani Chitnis, Ashka Shah, Sidharth Addepalli, Ria Paradkar, Rishika Chavali, Hana Mir, Anna Zheng, Lydia Steenman, Hannah Shorrock, Andrew Berglund, Vinata Vedam-Mai, Damian Shin

**Affiliations:** 1 Department of Neuroscience and Experimental Therapeutics, Albany medical College, 47 New Scotland Ave, Albany, NY 12208 USA; 2 Department of Neurosurgery, University of Florida, Gainesville, FL USA; 3 Department of Biology and RNA Institute, State University of New York at Albany, Albany, NY USA; 4 Department of Anatomical Sciences and Neurobiology, University of Louisville, 511 S. Floyd St. Room 111, Louisville, KY 40202 USA

**Keywords:** Spinocerebellar ataxia 1, Cerebellum, DigiGait, Hindlimb clasping, Behavioral testing, Sex differences

## Abstract

Spinocerebellar ataxia 1 (SCA1) is a rare autosomal dominant neurodegenerative disease characterized by impaired gait, coordination, and balance. SCA1 results from an expanded CAG repeat in the *Atxn1* gene, inducing protein aggregation and ultimately leading to the degeneration of cerebellar Purkinje cells. Clinical studies have shown that gait impairments, such as changes in stride length (SL), stride time, and stance phase, are seen in patients with cerebellar diseases. The SCA1^154Q/2Q^ mouse model reflects the longitudinal progression of SCA1 in humans, displaying motor incoordination, muscle atrophy, and cerebellar Purkinje cell degradation. In this study, we aim to characterize the progression of gait impairments that arise in the SCA1^154Q/2Q^ mouse model. The DigiGait™ system, which utilizes ventral plane imaging technology, was used to track gait parameters in SCA^154Q/2Q^ mice, beginning at 7 weeks of age until 42 weeks. Our data revealed that SCA^154Q/2Q^ males exhibited decreasing gait speeds beginning weeks 15–16 (*p* < 0.05), and SCA^154Q/2Q^ females showed gait speed declining as early as 9 weeks (*p* < 0.05). A decrease in SL was also found; these emerged at different time points in SCA1^154Q/2Q^ mice, ranging from weeks 14 to 32. Our data also suggest that SCA1 mice have decreased loading speed in hindlimbs with lower MAX dA/dt values at weeks 30 and 40 in both males and females (*p* < 0.01). Our characterization of this model establishes a framework for sex- and age-related differences, as well as a timeline of various gait performance metrics, which provides a foundation to test the efficacy of novel therapeutics.

**Supplementary Information:**

The online version contains supplementary material available at 10.1007/s12311-025-01910-2.

## Introduction

Spinocerebellar ataxias are a group of progressive, debilitating, and inherited neurodegenerative diseases caused by unstable expansions of CAG repeats, and spinocerebellar ataxia 1 (SCA1) is affected by instability in the *Atxn1* gene. The prevalence of SCA1 is higher in certain countries, with SCA1 making up 68% of families affected by SCA in Poland, 41% in Russia, 41% in South Africa, 34% in Serbia, 25% in Italy, and 20% in India [1]. SCA1 affects the brainstem, spinocerebellar tracts, and particularly, the Purkinje cells in the cerebellar cortex [2], due to the impairment of specific nerve fibers, resulting in the degeneration of the cerebellum. Clinically, patients present with impaired gait, coordination, and balance [3, 4]. Early symptoms of cerebellar dysfunction include speech difficulties, swallowing issues, and involuntary eye movements. Additional manifestations include pyramidal, extrapyramidal, and other neurological dysfunctions, as well as cognitive impairments. These deficits worsen over time, becoming markedly observable in advanced stages of the disease and eventually leading to mortality.

Mouse models have been an essential tool used to further our understanding of SCA1. The B05 mouse model was first described in 1995 and utilized a Pcp2/L7 promotor with an uninterrupted expanded polyglutamine tract (82Q) repeats to express human SCA1 coding region in the Purkinje cells of the mice [5]. These mice exhibited gait abnormalities, motor impairments, and reduced cage activity within five weeks of age. Later, the 154Q/2Q knock-in (SCA1^154Q/2Q^) mouse model was created to reflect the longitudinal progression of SCA1 in humans by displaying motor incoordination, cognitive deficits, muscle atrophy, and Purkinje cell loss [6]. Specifically, SCA1^154Q/2Q^ mice are reported to have 18.7% weaker grip strength compared to wild type (WT) counterparts along with impaired performance on rotarod tests, with variable onset, but as early as five weeks [6–8]. Cross sectional assessment using gait analysis software has found that SCA1^154Q/2Q^ mice display inconsistent gait patterns and reduced walking speeds compared to WT controls [7]. While detailed data on the cognitive and pathological changes exist in this mouse model, there is surprisingly sparse information about their gait impairment over time; further analysis of this animal model will allow researchers to track phenotypic improvements as novel therapeutics emerge.

Gait abnormalities present as the first symptoms in 80% of ataxias, including in 92% of SCA1 patients clinically [9]. A systematic review of gait characteristics associated with cerebellar ataxias found that patients displayed reduced walking speed, cadence, step length, stride length (SL), and swing phase [10]. Research studies utilizing wearable sensors have shown that clinical scales, such as Scale for Assessment and Rating of Ataxia (SARA), Brief Ataxia Rating Scale (BARS2), and Berg Balance Scale (BBS), are strongly correlated with various changes in gait, including SL, stride time, and stance phase, and difficulty controlling truncal oscillation, which can lead to balance and postural issues in patients with SCAs [11, 12]. Overall, spatial and temporal gait metrics have been used to refine our understanding of gait abnormalities in several disease pathologies, including mouse models.

To test gait abnormalities in our mouse models, we employ DigiGait™, which is a simple, sensitive, and objective method to evaluate functional deficits in locomotion and limb coordination in mice with ataxias, and the data can then assess consistency with the desired patient phenotype. In this study, we aim to characterize the longitudinal changes in gait performance, hindlimb clasping behavior, body weight, and terminal brain weight in the SCA1^154Q/2Q^ animal model. With this data, we will be able to track alterations in the symptomatic behavior of these animals to reveal the underlying pathophysiology of SCA1 for target engagement when developing novel therapeutics spanning genetic, molecular, pharmaceutical, or neuromodulatory approaches, which are currently limited.

## Methods

### Animal Husbandry

**Fig. 1:**
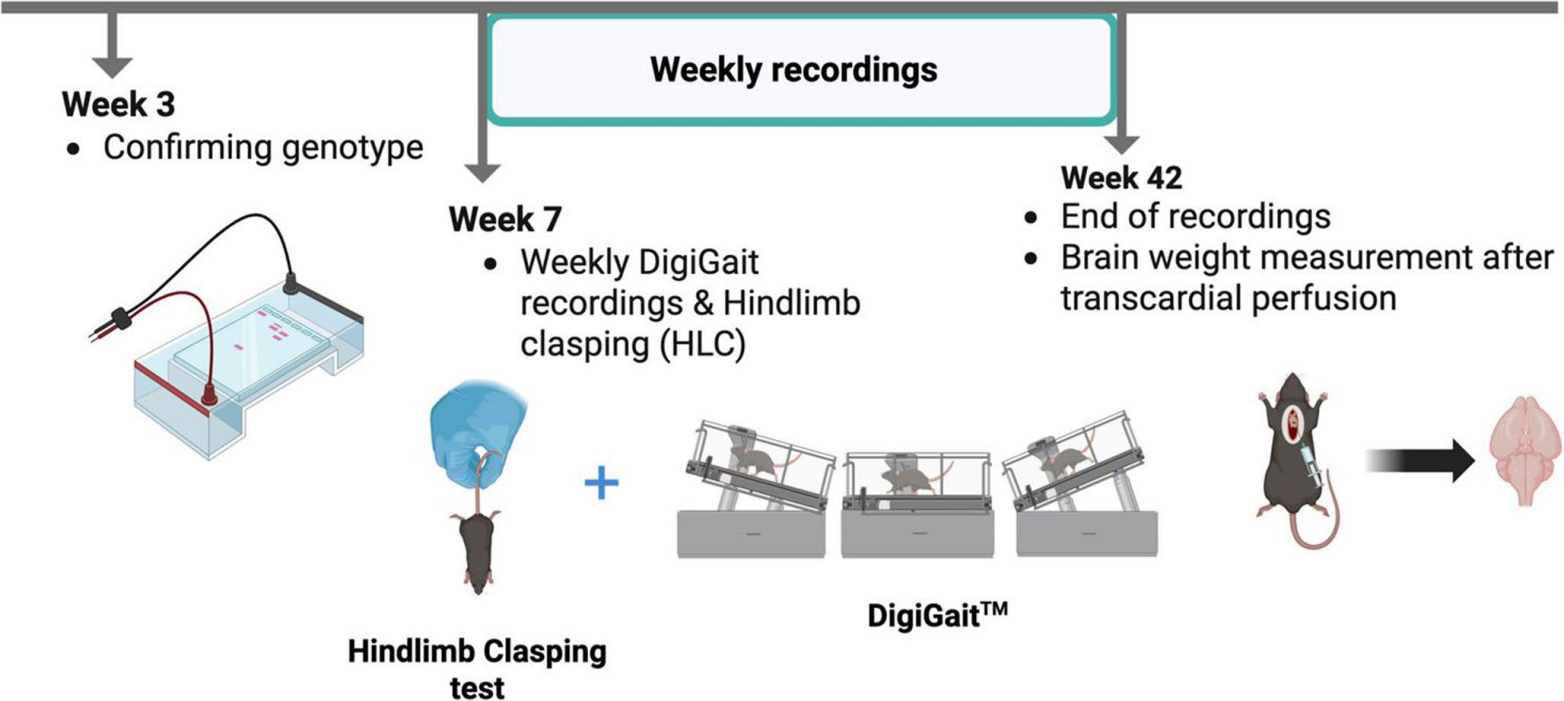
Overview of experimental timeline. Mouse genotypes were determined through PCR at 3 weeks of age. Starting at 7 weeks of age, weekly assessments were conducted, including examination of the HLC reflex, followed by gait analysis on the DigiGait™ treadmill. Weekly testing was concluded at 42 weeks of age, marked by brain weight measurement

All experiments were conducted in accordance with Albany Medical College (AMC) Institutional Animal Care and Use Committee and consistent with the National Institutes of Health Guide for the Care and Use of Laboratory Animals. Initially, two SCA1^154Q/2Q^ transgenic male mice (stock number #005601, B6.129S7-Atxn1^tm1Hzo^/J), originally purchased from Jackson laboratories (Bar Harbor, ME), were bred with WT C57BL/6J female mice; breeding pairs and colonies were housed in the Animal Resource Facility at AMC. After the initial generation, male breeders were chosen from litters at random after confirmation of at least 145 CAG repeats in the *Atxn1* gene. Female breeder mice were either from different litters than their paired male breeder or were C57BL/6J female mice purchased from Jackson Labs. All mice used for experiments were bred in the lab. Mice purchased directly from Jackson Labs were used solely for breeding, while control groups were composed of littermates to the experimental SCA1 mice. Mice were housed in Allentown housing cages with controlled conditions; temperature and humidity were set at 72 °F and 30–70% humidity, respectively, with a 12-hour light/dark cycle. Food and water were made available to all mice *ad libitum* throughout the experimental timeline (Fig. 1).

### Genotype

All animals were genotyped using NaOH digested tail tissue DNA obtained during weaning to identify the presence of heterozygous 154Q/2Q sequence. Polymerase chain reaction (PCR) was performed with SCA1 forward primer 5’GTG AGT TTG GGT CTG GCA TC3’ and SCA1 reverse primer 5’CCA AAA GTT AGG ATC ACA GCC C3’ (Invitrogen, Waltham, MA) with OneTaq Polymerase (New England Biolabs, Ipswich, MA).

To verify the number of CAG repeats and ensure the fidelity of repeats over generations, we performed PCR across the repeat on each new male mouse breeder (Supplementary Fig. 1). DNA was extracted from ear punch tissue using 0.3µg/mL Proteinase-K (Zymo Research, Irvine, CA). The PCR solution was created using SCA1-Rep Fw (5’ CGTGTACCCTCCTCCTCAGT 3’), SCA1-Rep-Rv (5’ ATTGCACAACCACCTGGGAT 3’), 1µM Phire Hot Start II DNA Polymerase (Thermo Fisher Scientific, Hanover Park, IL), 10µM Betaine (Thermo Fisher Scientific, Hanover Park, IL), 0.2µM 7-Deaza-dGTP (Sigma-Aldrich, St. Louis, MO), 2µM 10mM dNTPs (Thermo Fisher Scientific, Hanover Park, IL) [13]. Samples were sent for DNA sequencing after isolating DNA from PCR bands using the EZ-10 Spin Column DNA Gel extraction Kit (Bio Basic, Ontario, Canada). Samples were sent alongside nested primers Atxn-1-Nest-Fw (5’CTTACGCGGGCTTATCCCT3’) and Atxn1-Nest-Rv (5’CGTCTGATFFFFATGGAGGT3’).

### Gait Analysis

Gait performance was assessed in all animals each week for 35 weeks beginning at 7 weeks of age, with their weights noted prior to the treadmill session with the DigiGait™. This system employs Ventral Plane Imaging (VPI) technology using a high-speed video acquisition camera mounted below a transparent treadmill belt to continuously image the underside of walking animals to generate a ventral view of “digital paw prints.” The software identifies the paws utilizing their pink color. Animals were recorded on the treadmill for a minimum of four consecutive seconds of constant gait while walking at three different settings: Flat 0°, Incline 10°, and Decline 10°. The treadmill speed can vary from 0.1 to 99.9 cm/second, but we employed a maximum speed of 20 cm/s. If a mouse was unable to perform at this speed, the treadmill speed was set to the fastest speed the mouse could proficiently and continuously walk for four subsequent seconds. We identified each animal’s maximum speed after failing at 20 cm/s by conducting two trials. In the first trial, the animal was placed on the treadmill starting at 0 cm/s, and the speed gradually increased until the animal could no longer keep pace, indicated by backing into the end of the treadmill. Immediately after the first trial, we repeated this procedure with the maximum speed identified in the first trial to confirm successful performance.

DigiGait™ provides numerous metrics that can be tracked longitudinally, and all images obtained were digitized and imported for analysis with DigiGait™ video in-house imaging acquisition software (Mouse Specifics, Boston, MA) to analyze temporal and spatial gait metrics. Our initial selection of metrics was guided based on previously published studies and included: swing time, brake time, propulsion time, stance time, SL, stride width, MAX dA/dt, gait speed, animal width, animal length, and ataxia coefficient. After an initial sampling phase, we continued to monitor the metrics that showed significant differences – SL, gait speed, brake time, propulsion time, stance time, and MAX dA/dt.

In the sampling phase, we found that the onset of some gait metrics in SCA1^154Q/2Q^ mice emerged inconsistently. For instance, a significant difference in one metric may be observed in week 27, but not the following week, and then was significant again in week 29. This variability complicated the efforts to define a clear point of symptom onset. To address this, we defined the earliest timepoint of symptom emergence as the observance of a symptom without a loss of statistical difference for greater than two weeks.

### Hindlimb Clasping Behavior

In addition to DigiGait™ metrics, we also reported the emergence and severity of hindlimb clasping behavior (HLC), a common hallmark of cerebellar dysfunction. HLC was recorded by suspending the animal for 10 s by the base of their tail; this was conducted weekly in conjunction with gait performance testing. Videos were scored independently by two blinded and one unblinded scorer using a rubric (Supplementary Fig. 2) spanning a scoring range from 0 to 4, which we adapted from elsewhere [14]. Scoring was based on the following criterion: 0 = Hindlimbs are abducted away from the abdomen (> 50% of time), 1 = Right or left hindlimb adduct toward the abdomen (≥ 50% of time), 2 = Bilateral partial adduction of hindlimbs toward the abdomen (≥ 50% of time), 3 = Bilateral adduction of hindlimbs toward the abdomen (≥ 50% of time) with the addition of hindlimb crossing, and 4 = Bilateral full clasping (touching) the abdomen (≥ 50% of time).

### Brain Weights

At 42 weeks of age, animals were anesthetized using an intraperitoneal injection (IP) of urethane (Sigma-Aldrich, St. Louis, MO, 1.2–1.5 g/kg) and then euthanized with a double thoracotomy. Afterwards, a trans-cardiac perfusion was conducted using 10 mL of heparin (Medline, Northfield, IL) followed by 10 mL of paraformaldehyde (Santa Cruz Biotechnology, Dallas, TX). The brain weight was measured directly after extraction.

### Statistics

Statistical analysis was conducted using GraphPad Prism (Ver 9.2.1, GraphPad software, Boston MA). Normality was assessed with a Shapiro-Wilk test, and a parametric test was performed with a mixed-effects model (REML) with Fisher’s LSD multiple comparison test for all data sets except for hindlimb clasping behavior where we performed a Kruskal-Wallis non-parametric test with Dunn’s multiple comparison test. Specific time points at 8, 20, 30, and 40 weeks of age were analyzed to coincide with the lab-determined four physically discernible stages our SCA1 mice exhibited in the longitudinal study. All data points were presented as mean +/- standard error of mean (SEM).

## Results

### Longitudinal Phenotypic Changes

**Fig. 2:**
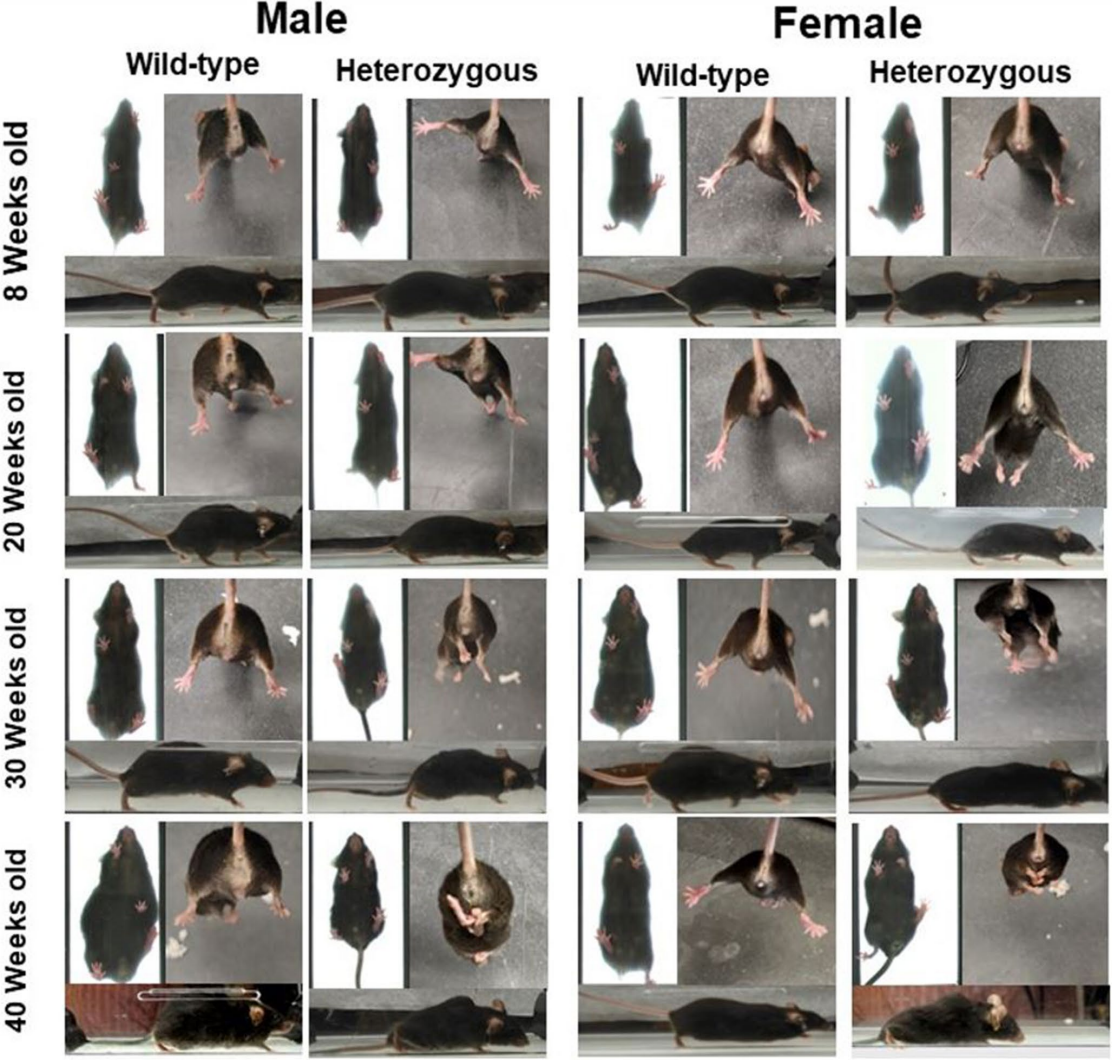
Phenotypes of wild type C57BL/6J and heterozygous SCA1^154Q/2Q^ mice tracked through disease stages. During the pre-symptomatic stage of SCA1, heterozygous male and female mice present a phenotype similar to their wild type littermates. At the onset of SCA1, around 20 weeks old, mice begin to show early signs of hindlimb clasping. When heterozygous mice reach 30 weeks old, moderate disease symptoms show with mice expressing kyphosis, muscle wasting, a shortening of body length, and a lowering of stature. Nearing the end of the disease, at 40 weeks old, mice show severe disease phenotypes, including severe decrease in overall gate speed, muscle tone, and body weight along with kyphosis, hindlimb clasping, and hindquarter dragging

Phenotypic changes in animals were tracked over time (Fig. 1). Our findings were characterized by dividing the symptomology timeline into four stages (Fig. 2). These stages were largely based on the development of kyphosis in the spine of SCA1^154Q/2Q^ mice: pre-symptomatic (0–8 weeks of age), disease onset (9–20 weeks of age), moderate symptomology (20–30 weeks of age), and severe symptomology (30–40 weeks of age).

At 8 weeks of age, SCA1^154Q/2Q^ mice were visually similar to their wild type of littermates and did not exhibit any hindlimb clasping behavior. Between 9 and 20 weeks of age, SCA1^154Q/2Q^ mice did not present with kyphosis but began to show subtle signs of disease progression, as seen with onset of body weight changes and subtle impairments noted in gait analysis. SCA1^154Q/2Q^ mice from 20 to 30 weeks of age displayed moderate disease symptomology in both male and female mice, with the emergence of kyphosis and a lowering of their posture. At 40 weeks of age, both sexes exhibited severe disease symptomology with marked kyphosis, hindlimb drag, shuffling gait, and diminutive appearance, plausibly from muscle wasting.

### Body Weight

**Fig. 3:**
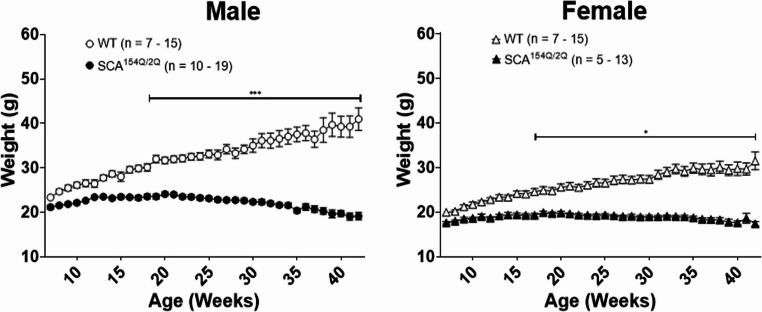
Weight differences between wild type C57BL/6J and heterozygous SCA1^154Q/2Q^ mice. Weights of SCA1^154Q/2Q^ and wild type mice were collected weekly from ages 7–42 weeks and display a separation between wild type and heterozygous mice beginning around week 7. A mixed effects analysis with repeated measures shows a significant difference between SCA1^154Q/2Q^ and WT groups beginning at week 16 for males and week 15 for females. Asterisks indicate significance: (*) denotes *p* < 0.05, (***) denotes *p* < 0.001

WT and SCA1^154Q/2Q^ groups showed differences in body weight at 16 weeks of age for males (*p* < 0.001) and week 15 for females (*p* < 0.05), which continued throughout the experimental timeline (Fig. 3). At week 16, SCA1^154Q/2Q^ males weighed 23.4 ± 0.4 g (*n* = 14) in comparison to WT males that weighed 29.6 ± 0.7 g (*n* = 12). Additionally, weight changes were indicated to be significantly different between WT and SCA1^154Q/2Q^ males over time (genotype x time: *F*_*35*, 842_ = 14.5, *p* < 0.0001) (Fig. 3 left). By 40 weeks of age, male SCA1^154Q/2Q^ and WT mice weighed 19.7 ± 0.6 g (*n* = 12) and 39.3 ± 2.4 g (*n* = 11), respectively. For females, the weights for SCA1^154Q/2Q^ and WT mice at 15 weeks of age were 19.4 ± 0.4 g (*n* = 7) and 24.2 ± 0.5 g (*n* = 9), respectively. Similar to males, females also exhibited an interaction between genotype and time for weight changes as they aged (genotype x time: *F*_*35, 618*_ *= 6.1*, *p* < 0.0001) (Fig. 3 right). At week 40, SCA1^154Q/2Q^ females weighed 17.6 ± 0.4 g (*n* = 7), whereas WT females weighed 29.4 ± 1.5 g (*n* = 12) (*p* < 0.0001). A comparison between male and female SCA1^154Q/2Q^ groups revealed that female SCA1^154Q/2Q^ mice weighed less than their male cohorts from weeks 7–34 of age (*p* < 0.05). The final week at which a weight difference was observed was at 34 weeks of age, with SCA1^154Q/2Q^ males weighing 21.6 ± 0.5 g (*n* = 14), while SCA1^154Q/2Q^ females weighed 18.9 ± 0.3 g (*n* = 7) (*p* < 0.05). By the end of the experimental timeline, at 42 weeks of age, sex differences between SCA1^154Q/2Q^ mice were no longer observed, as SCA1^154Q/2Q^ males weighed 19.1 ± 0.8 g (*n* = 5) and females weighed 17.4 g ± 0.5 g (*n* = 11).

**Fig. 4:**
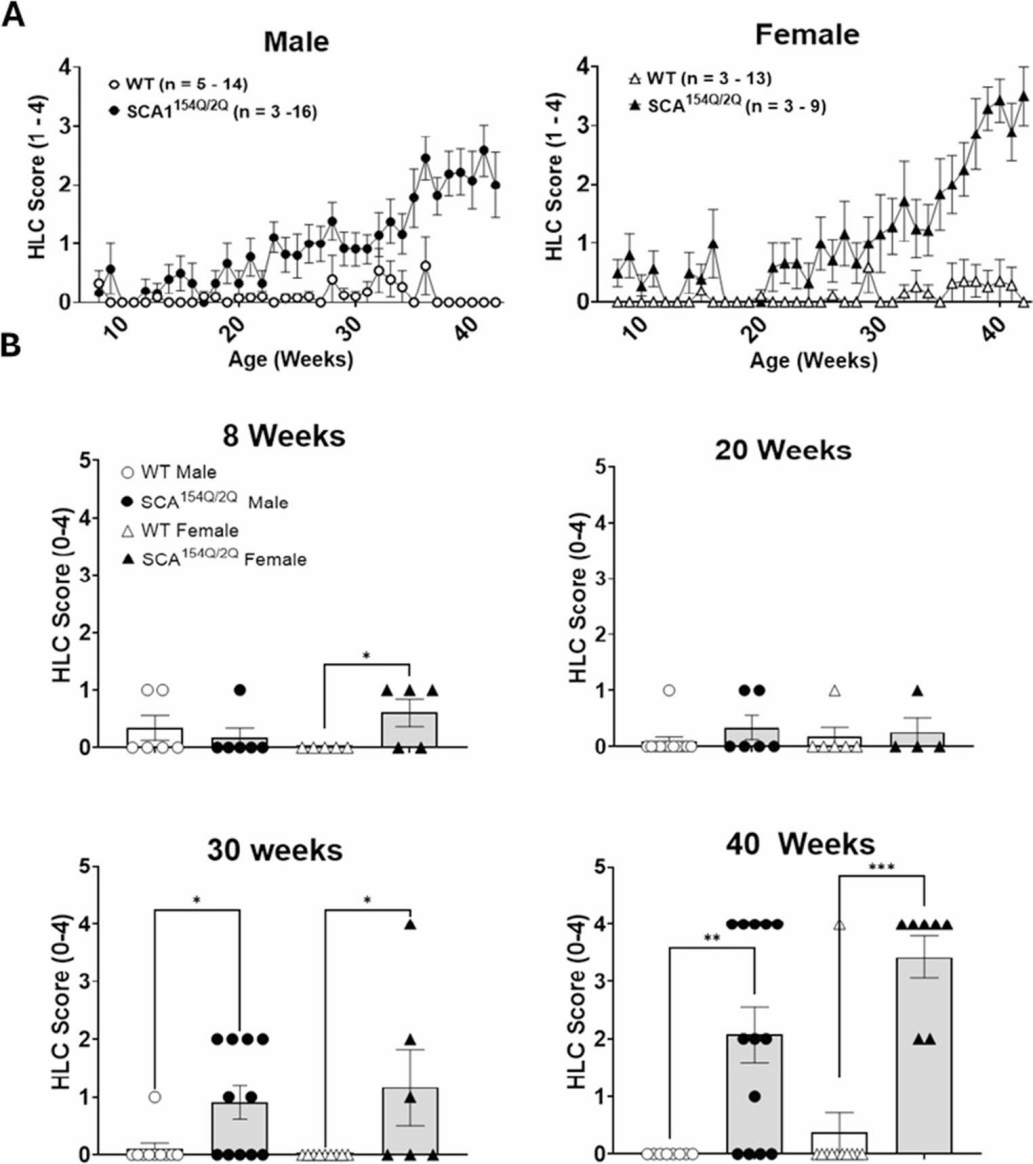
**a** The figure represents mean hindlimb clasping behavior scores for WT and SCA1^154Q/2Q^ from 8–42 weeks. **b** A Kruskal-Wallis test was used to compare differences between all groups at ages 8 weeks, 20 weeks, 30 weeks, and 40 weeks. Asterisks indicate significance: (*) denotes *p* < 0.05, (**) *p* < 0.01, and (***) denotes *p* < 0.001. Sample size for each timepoint for WT male, SCA1^154Q/2Q^ male, WT female, and SCA1^154Q/2Q^ female are as follows: 10 weeks (*n* = 6, 6, 5, 5), 20 weeks (*n* = 12, 4, 6, 4), 30 weeks (*n* = 10, 11, 9, 6), and 40 weeks (*n* = 8, 13, 11, 7)

### Hindlimb Clasping Behavior

At 8 weeks and 20 weeks of age, SCA1^154Q/2Q^ animals did not exhibit a difference in their HLC scores. Specifically, at 20 weeks of age, male SCA1^154Q/2Q^ animals had a score of 0.3 ± 0.2 (*n* = 4) whereas WT males had a score of 0.1 ± 0.1 (*n* = 12). Additionally, SCA1^154Q/2Q^ females had a score of 0.3 ± 0.3 (*n* = 6) and WT females had a score of 0.2 ± 0.2 (*n* = 4). At 30 weeks of age, the SCA1^154Q/2Q^ mice began to exhibit higher HLC scores in both males and females compared to their WT littermates (*p* < 0.05), and this difference persisted until 40 weeks of age (*p* < 0.05), with higher mean scores in both sexes (Fig. 4B). At that time, WT males had a score of 0.0 ± 0.0 (*n* = 8), which contrasted with SCA1^154Q/2Q^ male mice who had a score of 2.1 ± 0.5 (*n* = 13). Female SCA1^154Q/2Q^ mice at the same time point had a score of 3.4 ± 0.4 (*n* = 4), while WT females had a score of 0.4 ± 0.4 (*n* = 6).

### Gait Metrics

#### Animal Numbers

This experiment was conducted using multiple cohorts with variances of mouse numbers noted during the experimental timeline. Some mice were precluded from the data set if they refused to move on the treadmill in a specific week, after losing 20% of their body weight, or in rare cases, mortality. For the former, if a mouse was resistant to performing on the treadmill, they were tested the following week as part of the longitudinal study and only excluded from the study if the uncooperative behavior persisted for multiple and concurrent weeks. Higher animal cohorts were intentionally added in the later weeks as SCA1 symptomology and exclusion criteria became more prevalent. The n-values for all DigiGait™ metrics are provided in Table 1.

**Table 1:**
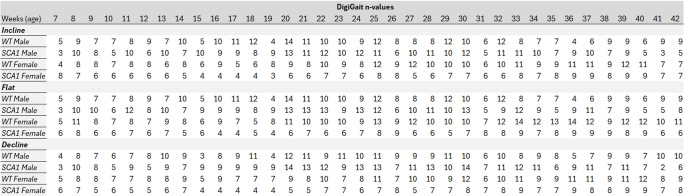
Index of sample sizes for each week of the 35 weeks of all DigiGait™ variables (± 2), when mice are 7 to 42 weeks of age. Varied numbers arise from animals refusing to perform at certain weeks, exclusion due to animal weight loss, or animal death

#### Gait Speed

**Fig. 5:**
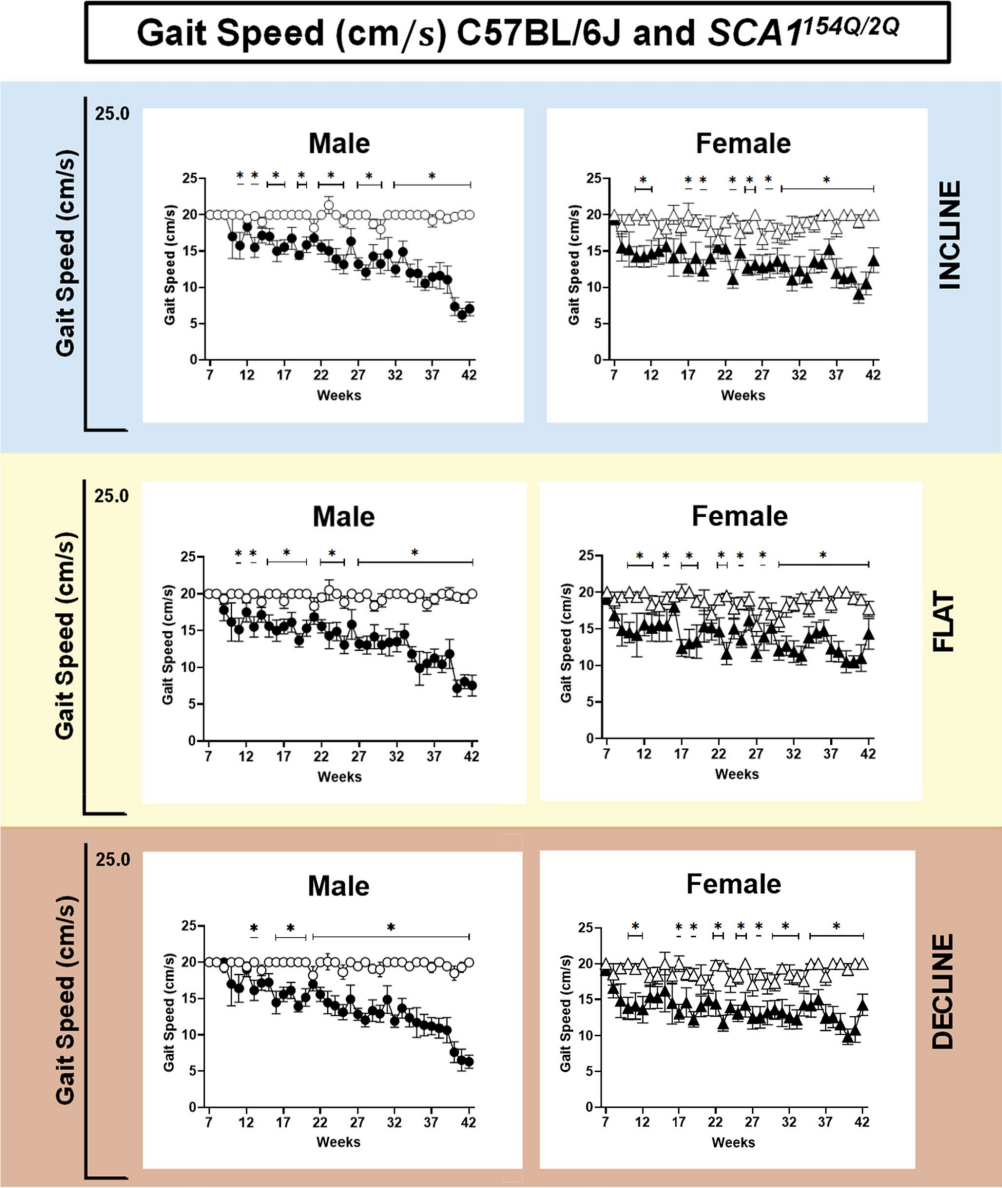
Gait speed of wild type C57BL/6J and heterozygous SCA1^154Q/2Q^ male and female mice on incline, flat, and decline angles. The side Y-axis shows an enlarged view of value range (0–25 cm/s). The X-axis ranges from the ages of weeks 7–42. A mixed effects analysis with repeated measures was conducted between the SCA1^154Q/2Q^ and WT groups. Asterisks indicate significance: (*) denotes *p* < 0.05

As stated in Sect. 1.3, the maximum speed was set at a baseline of 20 cm/s and then decreased based on the capability of the mouse; this results section details the variances in gait speed over time. SCA1^154Q/2Q^ males were observed to begin performing at decreasing speeds in all three angle settings at approximately 15 to 16 weeks of age. This decrease was more pronounced over time for all three angles (Fig. 5).

As they walked at a declined angle (Fig. 5 bottom), SCA1^154Q/2Q^ and WT males showed comparable gait speeds until week 15. At 16 weeks of age, SCA1^154Q/2Q^ males began to display a decreased speed, which became more pronounced as the mice aged (Time x Genotype: *F*_*35,544*_ *= 5.2*, *p* < 0.0001). At this age, the gait speed of SCA1^154Q/2Q^ males was 14.4 ± 1.5 cm/s, whereas WT controls were performing at a higher gait speed of 20.0 ± 0.0 cm/s (*p* < 0.05). For SCA1^154Q/2Q^ females, decreased speeds were observed beginning at the age of 17 weeks; however, there was no effect of age on the declining gait speed. The gait speed of SCA1^154Q/2Q^ females at 17 weeks of age was lower at 13.1 ± 1.2 cm/s, in contrast to 20 ± 0.0 cm/s for WT controls. The slower speed of SCA1^154Q/2Q^ females was persistent at 40 weeks of age, where the gait speed for SCA1^154Q/2Q^ females and WT females was 9.9 ± 1.1 cm/s and 19.2 ± 0.8 cm/s, respectively (*p* < 0.0001).

SCA1^154Q/2Q^ males began running at slower gait speeds starting at 15 weeks of age in the flat angle (Fig. 5 middle), in which their speed was slower at 15.6 ± 1.5 cm/s compared to a speed of 20 ± 0.0 cm/s for WT males (*p* < 0.05). The gait speed of SCA1^154Q/2Q^ males continued declining with age (Time x Genotype: *F*_*35,544*_ *= 4.9*, *p* < 0.0001) as their gait speed at 40 weeks of age was 7.2 ± 1.1 cm/s, in contrast to WT males who continued to run at 19.6 ± 0.4 cm/s (*p* < 0.001). SCA1^154Q/2Q^ female mice began to run at slower gait speeds earlier than males, at 9 weeks of age. The gait speed at 9 weeks of age for SCA1^154Q/2Q^ females was 14.8 ± 2.2 cm/s compared to 19.4 ± 0.6 cm/s for WT females (*p* < 0.01). Like males, the declining effect of age on gait speed was also observed in SCA1^154Q/2Q^ females (Time x Genotype: *F*_*35,492*_ *= 1.7*, *p* < 0.01). At 40 weeks of age, the gait speed for WT female mice was maintained at 19.2 ± 0.8 cm/s, whereas SCA1^154Q/2Q^ females slowed to 10.5 ± 0.8 cm/s (*p* < 0.001).

SCA1^154Q/2Q^ males walking in an incline angle (Fig. 5top) began to show a declining gait speed of 17.0 ± 1.1 cm/s at 15 weeks of age compared to their WT counterparts, who performed at 20.0 ± 0.0 cm/s (*p* < 0.05). The gait speed of SCA1^154Q/2Q^ males demonstrated a negative effect of age on gait speed (Time x Genotype: *F*_*35,521*_ *= 4.8*, *p* < 0.0001), as their gait speed at 40 weeks of age was 7.04 ± 1.2 cm/s, while WT males displayed higher speeds at 19.7 ± 0.3 cm/s (*p* < 0.001). The gait speed for SCA1^154Q/2Q^ females slowed compared to WT females beginning week 23, with a speed of 11.2 ± 1.3 cm/s, contrasted to WT females who ran at 19.6 ± 0.3 cm/s (*p* < 0.001). Similar to SCA1^154Q/2Q^ males, their gait speed continued to decline throughout the experimental timeline, concluding at week 40 (Time x Genotype: *F*_*35,447*_ *= 1.6*, *p* < 0.05), as SCA1^154Q/2Q^ females had a gait speed of 9.1 ± 1.3 cm/s in comparison to 19.1 ± 0.9 cm/s for WT females (*p* < 0.001).

In summary, the SCA1^154Q/2Q^ group demonstrated a clear reduction in walking speed compared to WT controls, with sex- and angle- specific differences in onset. Males showed reduced speeds beginning at approximately 15–16 weeks across all three angles, whereas females exhibited earlier impairments in the flat angle at 9 weeks, and later in decline and incline angles at 17 weeks and 23 weeks of age, respectively.

#### Stride Length (SL)

**Fig. 6:**
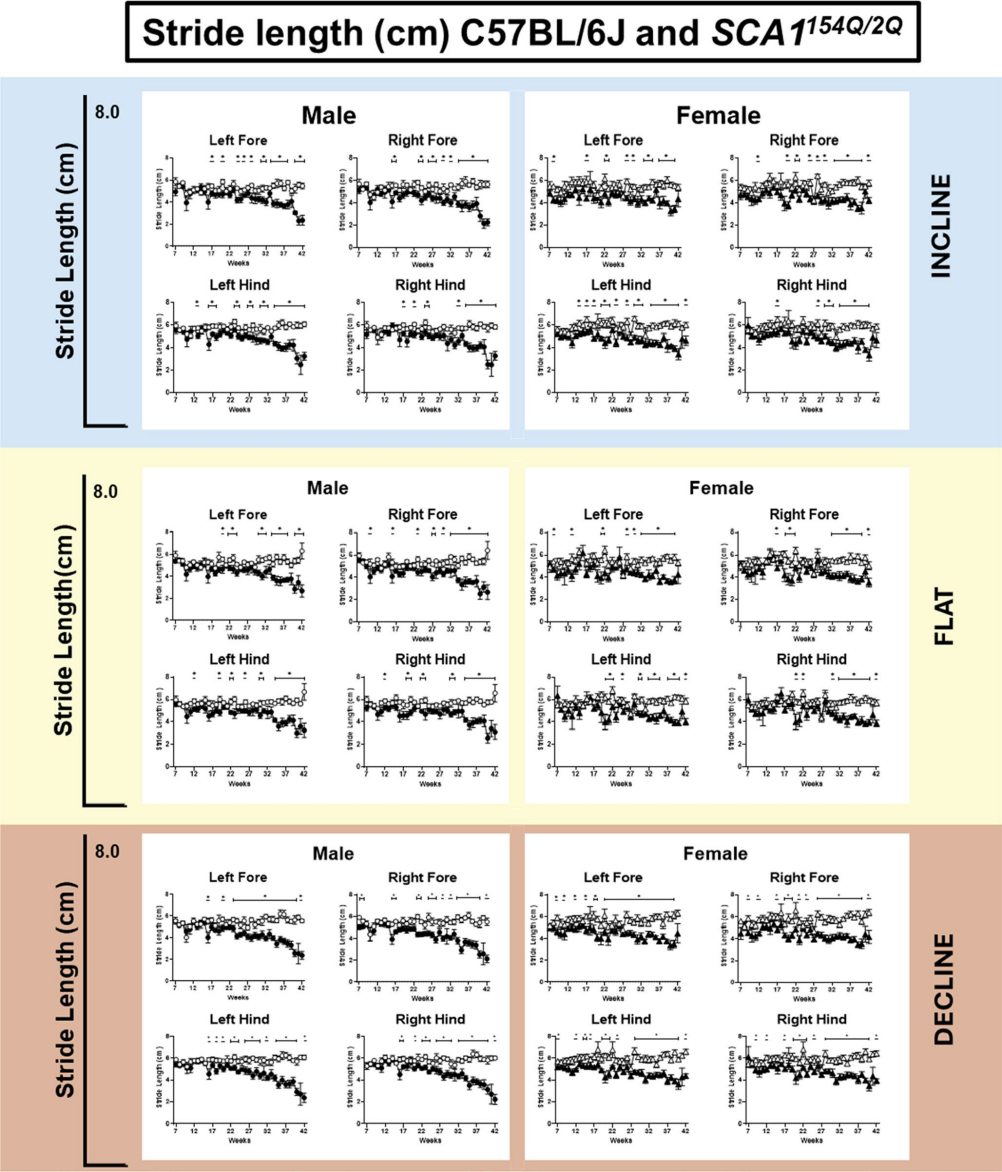
Stride length (SL) of wild type C57BL/6J and heterozygous SCA1^154Q/2Q^ male and female mice on incline, flat, and decline angles. The side Y-axis shows an enlarged view of value range (0–8 cm). The X-axis ranges from the ages of weeks 7–42. A mixed effects analysis with repeated measures was conducted between the SCA1^154Q/2Q^ and WT groups. Asterisks indicate significance: (*) denotes *p* < 0.05

SL is a measure of the distance between successive strides of the same paw. We found that SL decreased in all four limbs in SCA1^154Q/2Q^ animals in incline, flat, and decline angles (Fig. 6). Initially, SCA1^154Q/2Q^ males and WT males performed similarly in the left forelimb (LF) at a decline angle from the age of weeks 7 to 22. (Fig. 6 bottom). However, by 23 weeks of age, SCA1^154Q/2Q^ males had less SL in the LF than WT littermates, as SCA1^154Q/2Q^ males had a value of 4.9 ± 0.3 cm versus WT males at 5.7 ± 0.2 cm. Moreover, while WT males maintained their SL over time, SCA1^154Q/2Q^ males decreased SL as they aged (Genotype x Time: *F*_*35, 525*_ *= 4.2*, *p* < 0.0001), with a mean SL of 2.6 ± 0.3 cm at week 40 compared to 5.6 cm ± 0.4 in WT controls. The emergence of these changes varied between the forelimbs and hindlimbs in SCA1^154Q/2Q^ males. In heterozygous mice, similar to the LF, the right forelimb (RF) began demonstrating changes in SL at 23 weeks of age, whereas this change was seen earlier in the left hindlimb (LH) at 17 weeks of age and the right hindlimb (RH) at 16 weeks of age. Female SCA1^154Q/2Q^ animals began to exhibit decreased SL in their LF at week 28 with a mean of 4.5 cm ± 0.2, while WT female littermates had a SL value of 6.2 ± 0.3 cm. This decrease in SL in the SCA1^154Q/2Q^ female mice progressed as the animals aged (Genotype: *F*_*1,26*_ *= 65.8*, *p* < 0.0001; Time x Genotype: *F*_*35,445*_ *= 1.6*, *p* < 0.05), with these mice having a SL of 3.4 ± 0.4 cm at 40 weeks of age, but WT female mice had a value of 5.8 ± 0.2 cm in the LF.

In the flat angle (Fig. 6 middle), SCA1^154Q/2Q^ male mice began exhibiting decreased SL at 30 weeks of age, with the LF at a value of 4.3 ± 0.3 cm, while WT males had a SL value of 5.5 ± 0.4 cm (*p* < 0.05). As SCA1^154Q/2Q^ males aged, their SL further decreased (Time x Genotype: *F*_*35,550*_ *= 2.7*, *p* < 0.001), demonstrated by their week 40 values at 2.8 ± 0.4 cm, while WT males scored 5.4 ± 0.3 cm (*p* < 0.0001). The RF, LH, and RH also exhibited a decreased SL emerging at weeks 27 to 30 and were significant for the effect of time and genotype. The emergence of a decrease in SL was also variable among the different limbs in SCA1^154Q/2Q^ females. It first emerged in the LF at 28 weeks, followed by the LH at 29 weeks, 30 weeks in the RH, and 32 weeks in the RF. At 28 weeks, SCA1^154Q/2Q^ had a SL for the LF of 4.8 ± 0.3 cm, whereas WT female mice had a SL value of 5.8 ± 0.3 cm, (*p* < 0.05). The SL in SCA1^154Q/2Q^ females continued to decline over time (Time x Genotype: *F*_*35,492*_ *= 1.7*, *p* < 0.01), with a SL value of 3.6 ± 1.2 cm at week 40 in the LF, which differs from 5.5 ± 0.2 cm (*p* < 0.001) in the WT female group.

At the incline angle, a lower SL was seen in SCA1^154Q/2Q^ males compared to WT male controls at approximately 23 to 24 weeks of age in the LF, RF, and LH (Fig. 6 top). This decrease was observed later in the RH, at 32 weeks of age. Beginning at 24 weeks of age, in the LF, the SL for SCA1^154Q/2Q^ males was 4.2 ± 0.3 cm, which was lower than WT males who had a SL of 5.3 ± 0.1 cm, (*p* < 0.01). By week 40, this difference was even greater (Time x Genotype: *F*_*35,509*_ *= 2.8*, *p* < 0.0001), with a SL for SCA1^154Q/2Q^ males at 3.0 ± 0.1 cm contrasted to 5.2 ± 0.4 cm for WT males. SCA1^154Q/2Q^ females exhibited decreasing SL beginning week 28 in the LF, in which the mean SL for SCA1^154Q/2Q^ females was 4.4 ± 0.2 cm, in comparison to 5.9 ± 0.2 cm for WT females (*p* < 0.01). This difference continued throughout the rest of the project, where the SL mean at 40 weeks of age for SCA1^154Q/2Q^ females was 3.3 ± 0.4 cm but was 5.6 ± 0.2 cm in WT females (*p* < 0.01). The variances in SL between SCA1^154Q/2Q^ and WT females also differed between limbs with the earliest emergence in the LH at 14 weeks of age, followed by the RF at 20 weeks of age, and RH at 26 weeks of age. Additionally, while in SCA1^154Q/2Q^ females the hindlimbs displayed a shortening of SL as they aged, RH (Time x Genotype: *F*_*35,439*_ *= 1.6*, *p* < 0.05) and LH (Time x Genotype: *F*_*35,441*_ *= 1.8*, *p* < 0.05), this was not consistent in the forelimbs. This disparity between forelimbs and hindlimbs was not seen in SCA1^154Q/2Q^ males.

Together, these data demonstrate a consistent difference in stride length between SCA1^154Q/2Q^ and WT animals, and most changes emerged during the moderate symptomology stage, with some exceptions noted. Additionally, there were minimal sex differences, though there were some observed in the incline angle.

#### MAX dA/dt

As per Mouse Specifics Inc., MAX dA/dt is a calculated value that is determined by the maximal rate of change of paw area in contact with the treadmill during the braking phase to measure the ability of the animal to decelerate.

**Fig. 7:**
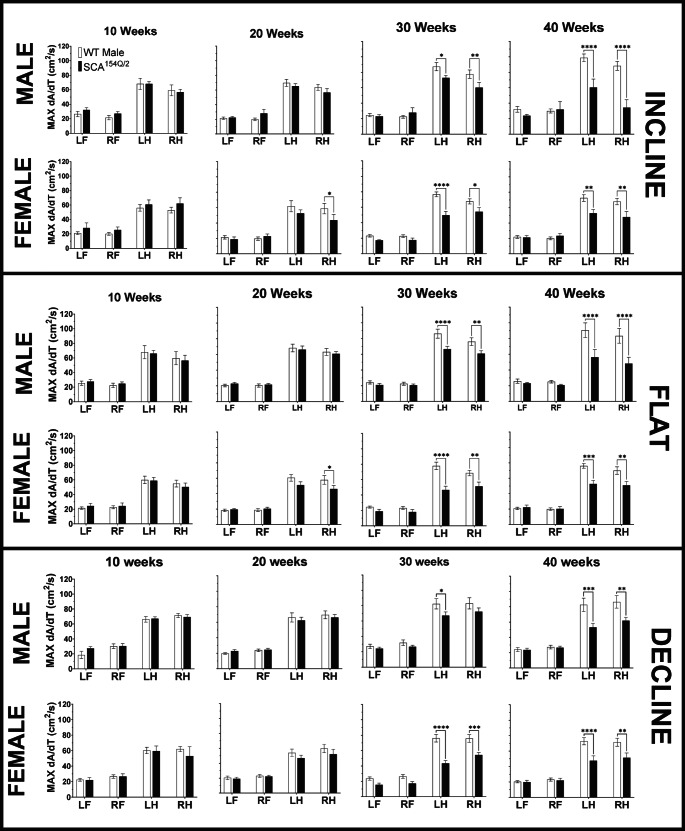
MAX dA/dt (cm^2^/s) of wild type C57BL/6J and heterozygous SCA1^154Q/2Q^ mice. The side Y-axis shows an enlarged view of value range (0-120 cm^2^/s). The X-axis shows the comparison between WT and SCA1^154Q/2Q^ LF, RF, LH, and RH limbs. A two-way ANOVA was conducted between the SCA1^154Q/2Q^ and WT groups. Asterisks indicate significance: (*) denotes *p* < 0.05, (**) denotes *p* < 0.01, (***) denotes *p* < 0.001, and (****) denotes *p* < 0.0001

SCA1^154Q/2Q^ males exhibited a decreased MAX dA/dt in both their LH and RH when compared to their WT counterparts at 40 weeks in the decline angle (Fig. 7 bottom). This decrease was only present in the LH in males at 30 weeks. At 40 weeks, the MAX dA/dt score in the LH was decreased for SCA1^154Q/2Q^ males (53.2 ± 5.1 cm^2^/s), compared to WT males (82.6 ± 8.7 cm^2^/s) (*p* < 0.0001). This decrease was also demonstrated in the RH, in which the MAX dA/dt value for SCA1^154Q/2Q^ males was 61.8 ± 4.4cm^2^/s compared to WT males at 86.4 ± 8.1cm^2^/s (*p* < 0.01). This trend was consistent in both hindlimbs of SCA1^154Q/2Q^ females at 30 and 40 weeks compared to their WT counterparts. At 40 weeks, the MAX dA/dt for SCA1^154Q/2Q^ females was 7.5 ± 6.3cm^2^/s in the LH and 51.3 ± 6.2cm^2^/s in the RH. WT females had a higher MAX dA/dt score of 72.7 ± 4.6cm^2^/s in the LH (*p* < 0.0001) and 71.5 ± 5.2cm^2^/s in the RH (*p* < 0.001).

The flat angle also demonstrated similar MAX dA/dt patterns compared to the decline angle, in which the RH and LH at 30 and 40 weeks were significantly different between genotypes (Fig. 7 middle). The MAX dA/dt in both LH and RH was decreased in SCA1^154Q/2Q^ males at 40 weeks (*p* < 0.001). The MAX dA/dt in the LH was 56.3 ± 9.6cm^2^/s, whereas WT males had a value of 90.6 ± 9.3cm^2^/s. In the RH, the MAX dA/dt for SCA1^154Q/2Q^ males was 48.1 ± 8.1cm^2^/s while WT males scored 83.4 ± 9.3cm^2^/s. In females, SCA1^154Q/2Q^ mice also showed decreased MAX dA/dt scores at 30 and 40 weeks. The MAX dA/dt at 40 weeks in the LH for SCA1^154Q/2Q^ females was 52.7 ± 4.5cm^2^/s, but only 76.0 ± 3.0 cm^2^/s in WT females (*p* < 0.001). In the RH, SCA1^154Q/2Q^ females had a MAX dA/dt of 50.7 ± 5.2cm^2^/s, and WT females had a score of 70.0 ± 4.9 cm^2^/s (*p* < 0.01).

There were also evident hindlimb MAX dA/dt impairments of SCA1^154Q/2Q^ male mice while walking at an incline angle at 30 and 40 weeks (Fig. 7 top). SCA1^154Q/2Q^ males at 40 weeks had a MAX dA/dt of 60.4 ± 10.8cm^2^/s, while WT males had a higher value of 98.8 ± 4.9cm^2^/s in the LH (*p* < 0.0001). In the RH, the MAX dA/dt for SCA1^154Q/2Q^ males and WT males were 34.3 ± 10.2cm^2^/s and 88.3 ± 5.7cm^2^/s, respectively. This was also consistent in females, as SCA1^154Q/2Q^ females demonstrated a decreased MAX dA/dt at 30 and 40 weeks. The MAX dA/dt in the LH of SCA1^154Q/2Q^ females was 52.7 ± 5.0cm^2^/s compared to WT females who displayed a MAX dA/dt of 98.8 ± 4.9cm^2^/s (*p* < 0.01). In the RH, SCA1^154Q/2Q^ females had a MAX dA/dt of 47.8 ± 7.3cm^2^/s compared to 98.8 ± 4.9cm^2^/s in WT females (*p* < 0.01).

SCA1^154Q/2Q^ mice were observed to have impaired deceleration at weeks 30 and 40 in the hindlimbs for all topographies, except for the RH of male SCA1^154Q/2Q^ mice in week 30 of the decline angle. There was no difference seen in the forelimbs of WT and SCA1^154Q/2Q^ groups (Fig. 7).

#### Brake time

**Fig. 8:**
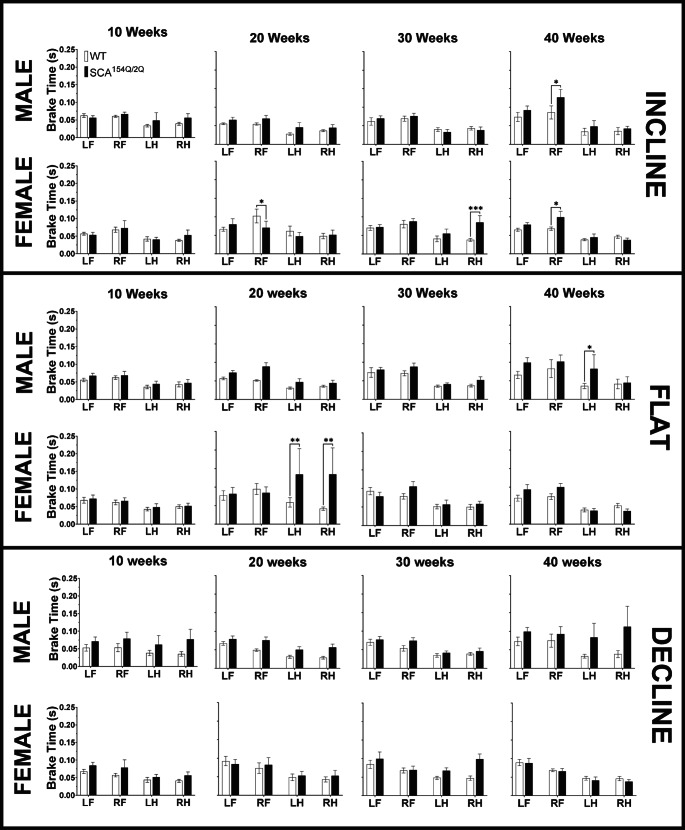
Brake time(s) of wild type C57BL/6J and heterozygous SCA1^154Q/2Q^ mice. The side Y-axis shows an enlarged view of value range (0.00–025 s). The X-axis shows the comparison between WT and SCA1^154Q/2Q^ LF, RF, LH, and RH limbs. A two-way ANOVA was conducted between the SCA1^154Q/2Q^ and WT groups. Asterisks indicate significance: (*) denotes *p* < 0.05, (**) denotes *p* < 0.01, and (***) denotes *p* < 0.001

Brake time is a temporal gait parameter that measures the difference between the initial time that the paw touches the ground and the point of maximal contact. SCA1^154Q/2Q^ mice exhibited an increased brake time in the RF at 40 weeks of age (*p* < 0.05) while running in an incline angle (Fig. 8). The SCA1^154Q/2Q^ males were recorded to have a longer brake time of 0.13 ± 0.02s, while WT males displayed a faster brake time at 0.09 ± 0.02s. Similarly, at 40 weeks of age, SCA1^154Q/2Q^ females were observed to have a higher brake time in the RF at 0.10 ± 0.02s compared to 0.07 ± 0.00s in WT females (*p* < 0.05).

#### Propulsion time

**Fig. 9:**
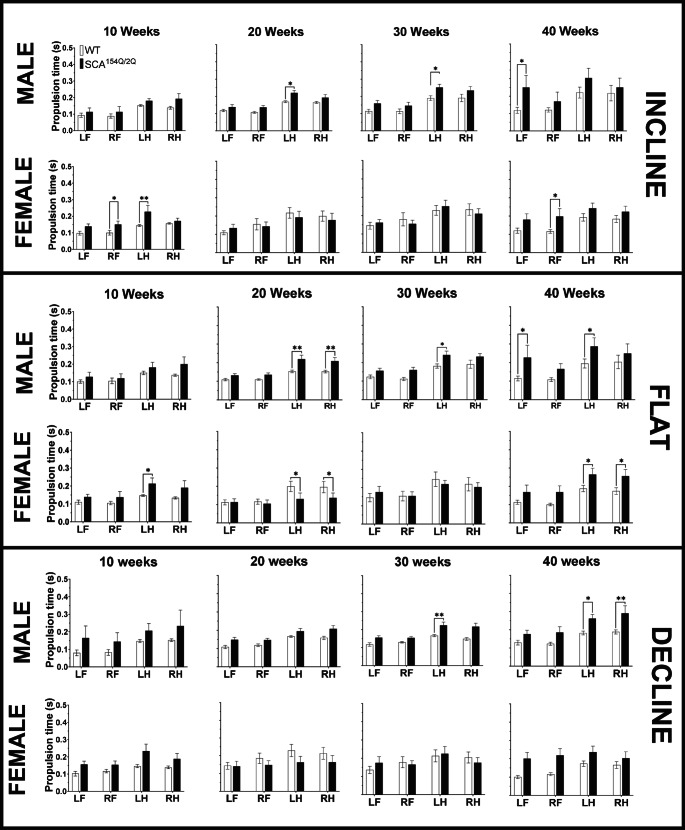
Propulsion time(s) of wild type C57BL/6J and heterozygous SCA1^154Q/2Q^ mice. The side Y-axis shows an enlarged view of value range (0.0–0.5 s). The X-axis shows the comparison between WT and SCA1^154Q/2Q^ LF, RF, LH, and RH limbs. A two-way ANOVA was conducted between the SCA1^154Q/2Q^ and WT groups. Asterisks indicate significance: (*) denotes *p* < 0.05, and (**) denotes *p* < 0.01

Propulsion time reflects the time between the maximal contact of the paw to the ground until the moment the paw is lifted from the ground. In the flat angle, beginning at 20 weeks of age, SCA1^154Q/2Q^ males demonstrated a longer propulsion time in the LH, 0.22 ± 0.02s, compared to WT males, 0.153 ± 0.01s (*p* < 0.01), which continued throughout the experimental timeline (Fig. 9 middle). This difference was consistent at 40 weeks of age, with higher propulsion times in SCA1^154Q/2Q^ males compared to WT males, with times of 0.29 ± 0.05 and 0.20 ± 0.02, respectively (*p* < 0.05). Additionally, at 40 weeks of age, the LF of SCA1^154Q/2Q^ males also exhibited a delayed propulsion time at 0.23 ± 0.07s, in comparison to WT males at 0.11 ± 0.01s (*p* < 0.05). In SCA1^154Q/2Q^ females, a longer propulsion time was observed in both the LH and RH at 40 weeks of age (*p* < 0.05). The recorded propulsion times in the LH was 0.26 ± 0.03s and 0.26 ± 0.04 in the RH for SCA1^154Q/2Q^ females compared to times of 0.20 ± 0.02s and 0.20 ± 0.04s in the LH and RH, respectively, in WT females.

Male SCA1^154Q/2Q^ mice were noted to have an increased propulsion time in the LH beginning at 30 weeks of age, which continued until 40 weeks of age as they walked in the decline angle (Fig. 9 bottom). At 40 weeks of age, SCA1^154Q/2Q^ males had a longer propulsion time at 0.26 ± 0.02s compared to 0.18 ± 0.01s for WT males (*p* < 0.05). Additionally, at 40 weeks of age, a longer propulsion time was also noted in the RH limb of SCA1^154Q/2Q^ males at 0.29 ± 0.04s, whereas WT males had a propulsion time of 0.19 ± 0.01 (*p* < 0.05). Female mice displayed no genotypic differences on the decline angle.

SCA^154Q/2Q^ males demonstrated consistent increases in propulsion times in their LH starting at 20 weeks of age in the flat angle and 30 of age weeks in the decline angle. This contrasted SCA^154Q/2Q^ females who were found to have an increased propulsion time in the flat angle at 40 weeks for hindlimbs only (Fig. 9 middle).

#### Stance time

**Fig. 10:**
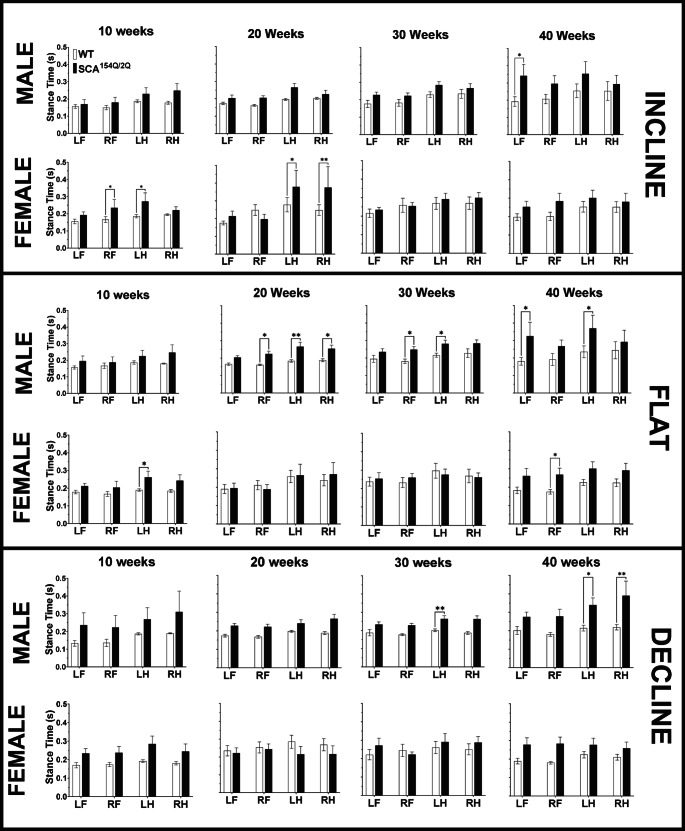
Stance time(s) of wild type C57BL/6J and heterozygous SCA1^154Q/2Q^ mice. The side Y-axis shows an enlarged view of value range (0.0–0.5 s). The X-axis shows the comparison between WT and SCA1^154Q/2Q^ LF, RF, LH, and RH limbs. A two-way ANOVA was conducted between the SCA1^154Q/2Q^ and WT groups. Asterisks indicate significance: (*) denotes *p* < 0.05, and (**) denotes *p* < 0.01

Stance time is the duration of time the paw is in contact with the belt. It is the sum of the brake and propulsion times. While running at a flat angle, it was observed that SCA1^154Q/2Q^ males demonstrated an increased stance time in the LH beginning at 20 weeks of age and reoccurred at 30 and 40 weeks of age as well (Fig. 10 middle). At 20 weeks of age, SCA1^154Q/2Q^ males had a longer stance time in the LH at 0.27 ± 0.03s, compared to WT males, who had a result of 0.18 ± 0.00s (*p* < 0.01). This increase was consistent at 40 weeks of age; SCA1^154Q/2Q^ males had a stance time of 0.37 ± 0.08, whereas WT males’ stance time was 0.24 ± 0.03 (*p* < 0.05). The increase in LH stance time was accompanied by a similar pattern in the RF of SCA1^154Q/2Q^ males at 20 and 30 weeks of age but was absent at 40 weeks. At 30 weeks of age, the LH stance time was 0.25 ± 0.02s for SCA1^154Q/2Q^ males compared to 0.18 ± 0.01s in their WT counterparts (*p* < 0.05). At 40 weeks of age, SCA1^154Q/2Q^ presented with an increased stance time in the LF in conjunction with the ipsilateral hindlimb. The stance time for WT males was lower at 0.18 ± 0.02s, whereas SCA1^154Q/2Q^ males had a stance time of 0.33 ± 0.08s (*p* < 0.05).

While walking at a flat angle, SCA1^154Q/2Q^ males showed consistent increase in stance time in the LH beginning week 20, but the same consistent genotypic effect was not observed in females. Furthermore, the incline and decline angles did not have consistent genotype or sex effects.

### Brain Weights

**Fig. 11:**
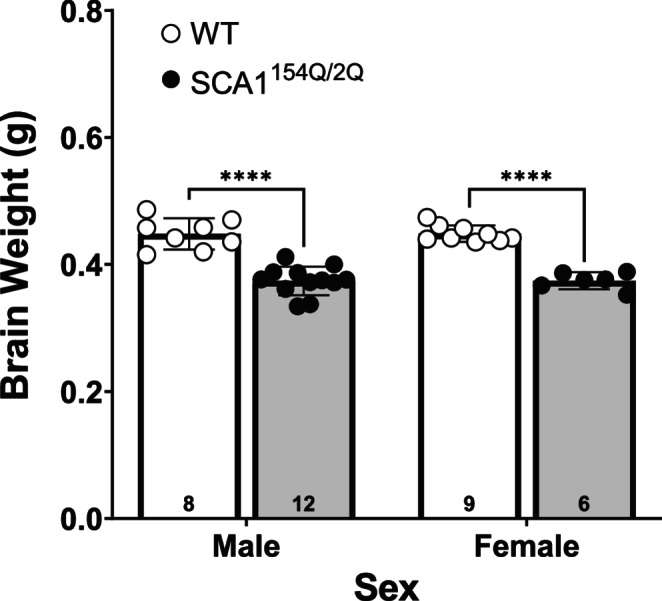
Perfused brain weights of wild type C57BL/6J and heterozygous SCA1^154Q/2Q^ mice. At the end of the test period of 42 weeks, mice were perfused trans-cardiac approach with heparin, followed by 4% PFA in saline. A two-way ANOVA showed that brains of WT male and female mice weighed more than their SCA1^154Q/2Q^ littermates, (****) denotes *p* < 0.0001. The sample size for each group is shown at the bottom of each bar

All brain weights were from 42 weeks of age. SCA1^154Q/2Q^ brains weighed less than WT brains in both males and females (*p* < 0.0001). In contrast, no differences were found between male and female groups (Fig. 11). SCA^154Q/2Q^ male brains weighed 0.374 g ± 0.006, in comparison to WT male brains that weighed 0.448 g ± 0.009. The brain weight of SCA1^154Q/2Q^ female was 0.374 g ± 0.005 in comparison to 0.449 g ± 0.004 for their WT counterparts.

## Discussion

We found that SCA1^154Q/2Q^ mice exhibited severe gait impairment as they aged, specifically decreased gait speed for all three walking angles, decreased SL in all four limbs, and impaired deceleration ability in their hindlimbs as indicated by a decreased MAX dA/dt. Additionally, non-gait symptoms were also present, including muscle wasting (as evidenced by genotypic weight differences), hindlimb clasping behavior, and brain atrophy (Table 2).

**Table 2:**
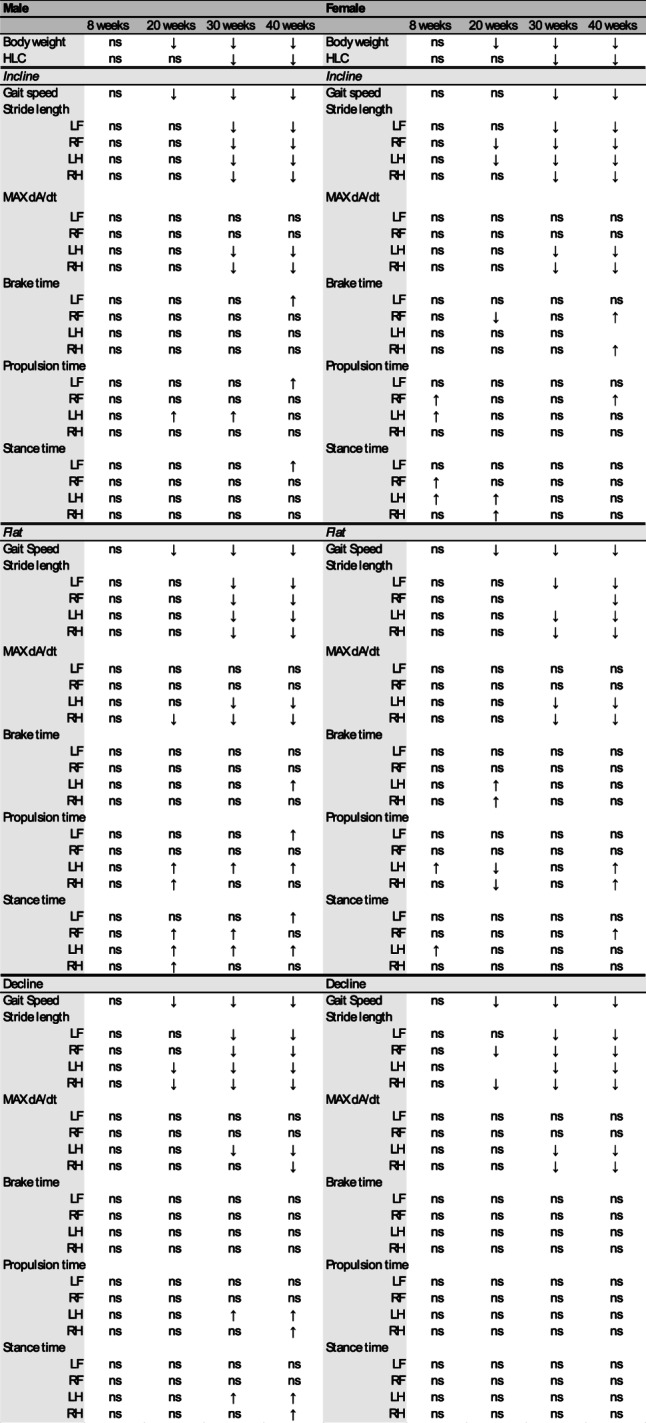
Summary of gait and locomotion metrics from WT and SCA1 male and female mice at four specific ages in our longitudinal study design. An arrow indicates significance, and the direction denotes increase or decrease; ns denotes ‘no significant difference’

We used physical appearance and overt gait changes as the key determinates for the four stages of disease in our mice models. In the first 8 weeks of life, SCA1^154Q/2Q^ mice did not exhibit any kyphosis or impaired gait performance, indicating a presymptomatic disease stage. Compared to their WT cohorts, SCA1^154Q/2Q^ animals began showing decreased gait speeds and the start of body weight changes around 8 to 20 weeks, so we characterized this time as disease onset. This progression aligned with other mouse model studies that found that although subtle rotarod impairment was seen as early as five weeks [6, 15], ataxia was generally observed at twelve weeks of age [15]. This symptom development was also comparable to the clinical presentation of SCA1 in humans, in which disease onset is often first characterized by slight gait difficulties [9]. For the moderate stage of ataxic symptomology, we observed the development of numerous gait changes across time and angles. SCA1 is associated with the fastest SARA progression compared to other spinocerebellar ataxias [16], and we found a rapid worsening of gait symptoms during 20-30 weeks of age. At around 30 weeks, SCA1^154Q/2Q^ mice began exhibiting muscle atrophy and kyphosis caused by a loss of muscle tone in spinal muscles [14], which is also associated with the clinical presentation of the disease [9]. Further, we observed increased scores in the HLC test, indicating marked cerebellar dysfunction, along with increased prominence of kyphosis and worsening gait symptoms at this time, with progressive worsening until the end of the study timeline, exclusion from the study as a result of greater than 20% loss of its body weight, or seldomly, death. The animals’ early exclusion and death is unsurprising considering SCA1 has the least favorable prognosis among the most common spinocerebellar ataxias [16]. We ultimately classified 30-40 weeks of age the severe disease stage.

As a result of this determination of the four stages of SCA1^154Q/2Q^ mice, we chose four evenly-spaced time points for statistical analysis throughout our longitudinal study to correspond with each of the stages. For instance, week 10 occurs in disease onset, week 20 in the moderate disease stage, week 30 in severe disease stage, and week 40 in end-of-life/severe stage. Therefore, swing time, brake time, propulsion time, stance time, and MAX dA/dt were all analyzed at these designated time points, while gait speed and stride length were analyzed every week as there were more apparent differences over time for these two variables.

In cerebellar ataxias, slower gait speeds, increased stance duration, and reduced SL are strategies used by patients as a compensatory mechanism for their ataxic gait [17, 18]. Our results demonstrate that these changes are also apparent in the SCA1^154Q/2Q^ mouse model, supporting its translational relevance for studying cerebellar gait dysfunction. The adoption of these compensatory patterns likely reflects impaired cerebellar coordination, in addition to neuropathic changes that are known to occur in the peripheral nervous system. In a study that investigated peripheral nerve involvement, abnormal electrophysiological findings were recorded in four out of five patients with SCA1 [19]. Specifically, three exhibited motor/sensory neuronopathy, while one presented with an axonal sensorimotor dying-back neuropathy. Consistent with these findings, histological and electrophysiological assessments of the peripheral nervous system in 25-week-old SCA1^154Q/2Q^ mice revealed demyelination and degeneration of spinal motor neurons, along with delayed nerve conduction from the spinal cord to the hindlimbs, and reduced muscle responsiveness [20]. Notably, these pathological features correspond with our observation of elevated MAX dA/dt scores in the hindlimbs of our SCA1^154Q/2Q^ mice at 30 and 40 weeks of age, which are an index of impaired deceleration during locomotion. These findings emphasize the multifactorial nature of motor dysfunction in SCA1 and demonstrate the ability of the SCA1^154Q/2Q^ model to capture cerebellar and peripheral degeneration.

We found a few differences with onset between sidedness of paw performance for some of the gait analysis variables. For instance, the four limbs had varying onset times for SL, with male SCA1 mice presenting with decreased SL at 23 to 24 weeks except for the right hindlimb that had an onset eight weeks later. While ataxic gait is generally symmetrical [21], asymmetrical gait differences have been reported in spinocerebellar ataxia 3 [22], which has a similar disease manifestation to SCA1 [23]. Researchers found that asymmetry in gray matter and cerebellum degeneration can affect gait asymmetry, especially in the early stages of the disease [22]. It is plausible that asymmetry in onset for SL in our SCA1^154Q/2Q^ mice stemmed from spatial and hemispheric Purkinje cell loss in the cerebellum or because of individual body differences [10]. Nonetheless, the appearance of asymmetry in gait variables in this study may warrant future investigation to reveal underlying processes for these observations.

Cell loss in the cerebellum may also result in the onset of SL differences between the forelimbs and hindlimbs. Stride length is comprised of two phases, the flexion and extension phase, and is affected by numerous factors including, but not limited to, leg length, speed, and cadence. Other studies that observed ataxic symptoms also found that hindlimbs were affected prior to the forelimbs [24, 25], which may correlate to the cerebellar atrophy often associated with increased HLC scores. Both forelimb and hindlimb motor function are controlled by the cerebellum, but the hindlimbs are more predominately affected by this brain structure [26, 27]. With that in mind, the disparity between forelimbs and hindlimbs is not a gait symptom that humans experience, given our bipedal locomotion, and there have been gait differences observed between the two species. Specifically, humans shorten their SL as they age, while mice often have a slower cadence [28]. Propulsion time and the flexion phase, when limbs leave the ground and joints flex, are highly related [29] and the increase of propulsion time in SCA1^154Q/2Q^ mice over time indicates that their cadence slows and their SL decreases. Additionally, our data may also provide evidence that the slower cadence of SL in mice stems from changes in the hindlimbs first, as we found SL decreased with age, but hindlimbs displayed a decrease approximately six weeks earlier than forelimbs in the decline angle. Future experiments may better tease apart the mechanics of SL to identify the underlying causes and relationships of the gait cycle phases during symptom progression.

Ataxic gait is the key symptom of SCA1 but is not the only one observed in our study. Body weight decline is associated with diseases caused by CAG repeat length, including SCA1 [30]. In mice, this symptom presents as a failure to gain weight [31] and kyphosis, both of which were present in our study. SCA1^154Q/2Q^ mice weighed significantly less than their WT cohorts as early as 15 and 16 weeks of age, for both female and male mice, respectively. The onset of this corresponded with the onset of gait speed, which occurred within the same few weeks. Gait difficulties are often one of the first symptoms of the clinical presentation of SCA1 [9], and decreasing body mass index is correlated with disease progression in clinical patients. The reason for weight loss is not clear in either humans or mice [30], but we posit it may be associated with muscle atrophy, which rapidly devolved in our study, as underscored by the emergence of kyphosis. Symptomology regression may deteriorate from a complex interaction and interdependence between muscle atrophy, weight loss, and gait symptomology.

While the mouse model used in our study was the SCA1^154Q/2Q^, there are other mouse models created to represent SCA1. The first model developed was the B05 model, which used a PCP2 promoter to express mutant ataxin-1 protein in the Purkinje cells of the cerebellum. These mice displayed gross locomotor instability, which led to frequent falls during ambulation [5]. However, this model only displayed symptomology associated with dysfunctional Purkinje and motor cells and generally lived a normal mouse lifespan [5, 6]. Therefore, the SCA1^154Q/2Q^ knock-in model was developed to create a more accurate genetic model, as it displayed longitudinal changes in weight, cognitive impairment, and impaired performance on the rotarod apparatus [6]. Our research expanded on previous findings regarding motor function by utilizing the DigiGait™ system instead of the rotarod. Other researchers observed that SCA1^154Q/2Q^ mice displayed an impaired performance beginning at five weeks on the rotarod [6], which is earlier than our findings with gait analysis. This may be because while the rotarod primarily challenges balance and coordination, it differs fundamentally from treadmill walking, which emphasizes rhythmic location. Balancing on the rotarod tends to be more demanding, often enabling earlier detection of motor dyscoordination. Gait analysis complements these assessments through a quantitative evaluation of specific abnormalities, providing deeper insight into locomotor impairments beyond general coordination deficits.

In order to understand the impact of human *Atxn1* on sex differences, a conditional knock-in model denoted as *f-ATXN*^*146Q*^ has 146Q repeats under the control of an endogenous mouse promoter and was created to display progressive SCA1 symptomology with sex differences. Female *f-ATXN*^*146Q*^ were observed to have better motor performance on rotarod and balance beam tests, as well as decreased weight loss and microglial activation compared to male *f-ATXN*^*146Q*^ mice [31]. In contrast, our study using the SCA1^154Q/2Q^ model revealed only subtle sex-based variations in symptom progression. Notably, female SCA1^154Q/2Q^ mice displayed earlier onset of gait impairment with reduced gait speeds at 9 weeks, whereas males only began showing slower speeds at 15 weeks. Additionally, male SCA1^154Q/2Q^ mice displayed an earlier onset of changes in propulsion time and stance time. This pattern differs from what was seen in the *f-ATXN*^*146Q*^ model, where males demonstrated an earlier onset of motor dysfunction on the rotarod [31]. Despite these differences, both *f-ATXN*^*146Q*^ and SCA1^154Q/2Q^ models exhibited a “failure to gain weight phenotype”. In the *f-ATXN*^*146Q*^ model, females failed to gain weight after 12–14 weeks, while males began to lose weight at 14 weeks, which was similar to SCA1^154Q/2Q^ males and females who reached their peak weights at 20 and 18 weeks of age, respectively. Given that there are subtle sex differences in SCA1, findings from both studies emphasize the importance of considering both sex and genetic context when interpreting phenotypic variability in these ataxic mice models.

Speed is a confounding variable known to affect several other metrics, such as stride length, stride frequency, swing time, and stance time [32, 33]. To account for this, researchers utilize several different methods, such as employing a constant predetermined speed, conducting subgroup analysis, or applying statistical models that adjust for gait velocity [34, 35]. While these methods enhance direct comparability across metrics, they also introduce limitations. For instance, post-experimental statistical adjustments can be time-intensive, which reduces the practicality of gait analysis as a rapid and scalable assessment tool. One of the primary advantages of analyzing gait speed at their natural limit is the ability to capture impairments as they emerge, regardless of pathological or compensatory causes. Controlling gait speed may inadvertently mask the evolution of disease-related gait changes, as underscored by our study demonstrating the appearance of decreasing speeds as an early indicator of disease progression.

One consideration to our design was that we did not investigate cognition and/or neurophysiological changes. Nonetheless, we did note severe brain atrophy in SCA1^154Q/2Q^ mice at 42 weeks of age, and research indicates people with SCA1 suffer from brain atrophy in multiple brain regions, including the basal ganglia, cerebellum, and hippocampus [36]. These areas are involved in motor function but also have a role in cognition. Future studies can investigate the specific neuronal pathways and brain areas affected by SCA1 utilizing a longitudinal study design to capture the data. Additional considerations to our research are the possible learning effects and physical tolls on the mice due to repeated use of the DigiGait™ system. The mice used the DigiGait™ system once a week for 35 weeks, and while the DigiGait™ system has previously been found to have a minimal learning component [37], this repeated use may have resulted in our experienced mice performing differently than mice at 42 weeks who had no previous experience. Furthermore, the once-weekly exercise may have had an indirect positive effect on the SCA1^154Q/2Q^ mice, especially considering previous research has found daily treadmill training increases neuron survival in the cerebellum [38]. Future research may use naïve mice to rule out these potential effects.

Overall, our data indicate long-term behavioral and physical changes to the SCA1^154Q/2Q^ mouse model and specify a complete timeline of SCA1 gait, hindlimb behavior, and physical appearance to give fundamental information not currently present in the literature. The DigiGait™ system provides multiple quantitative measurements to analyze gait in contrast to clinical gait assessment, which is often qualitative or at least somewhat based on subjective assessment [39]. While mouse gait may not be easily interpreted due to their quadrupedal stance compared to humans’ bipedal, our results provide a foundation of preclinical measurements that reflect the gait activity of people with SCA1. Future studies may apply these results to reflect human SCA1 symptomology so that physical and occupational therapies can be developed for any stage of the disease. These results provide more data on the SCA1^154Q/2Q^ mouse model and may serve as references to future therapies developed for the disease.

## Conclusion

Our study is the first to investigate the progression of gait ataxia and physical characterization of SCA1 in the SCA1^154Q/2Q^ mouse model. These mice had gait symptomology that worsened over time and a failure to gain weight phenotype, which is comparable to the progression of the disease in humans. Furthermore, while we did not find significant sex differences, we did find slight differences in the presentation of gait symptoms between the two sexes, which may lead to further investigations to tease apart the underlying mechanisms. Future studies will expand upon our foundation by exploring cognition, electrophysiology, and other potential symptomology that these mouse models present. These results provide a deeper understanding of the long-term development of the disorder in the SCA1^154Q/2Q^ mouse model and can serve as a foundational bedrock for future longitudinal exploration.

## Supplementary Information

Below is the link to the electronic supplementary material.

ESM 1 (DOCX 692 KB)

## Data Availability

Raw data sets generated during the current study are available from the corresponding author on reasonable request.

## Abbreviations

WT: Wild type
SCA1: Spinocerebellar ataxia 1
LF: Left forelimb(s)
RF: Right forelimb(s)
LH: Left hindlimb(s)
RH: Right hindlimb(s)
SL: Stride length
HLC: Hindlimb clasping
