# Supplementary material for: Longitudinal Study and Characterization of Gait Impairment in a Mouse Model of SCA1
Source: Cerebellum. 2025 Sep 18;24(6):157. doi: 10.1007/s12311-025-01910-2 (PMC12446116; doi:10.1007/s12311-025-01910-2)
Supplement: Supplementary file 1 — (DOCX 692 KB) [file 12311_2025_1910_MOESM1_ESM.docx]

**Supplemental figures**

**Supplemental figure 1.** Sanger sequencing results show 146 CAG repeats in breeder SCA1 male 683. a)

Zoomed in image of the CAG trace. The heightened peaks represent dominant nucleotide traces at each

base position: Cytosine (Blue), Adenine (Green), and Guanine (Black) b) Overview of the sequence of

146 CAG repeats, highlighted in blue.

**Supplemental figure 2.** Hindlimb clasping behavior scoring paradigm. The tail suspends mice, and a video is recorded for 10 seconds. Behavior is scored with the following paradigm. 0 = Hindlimbs are abducted away from the abdomen (> 50% of time), 1 = Right or left hindlimb adduct toward the abdomen (≥50%of time), 2 = Bilateral partial adduction of hindlimbs toward the abdomen (≥50% of time), 3 = Bilateral adduction of hindlimbs toward the abdomen (≥50% of time) with the addition of hindlimb crossing, and 4 = Bilateral full clasping (touching) the abdomen (≥50% of time)
